# All-optical polarization encoding and modulation by nonlinear interferometry at the nanoscale

**DOI:** 10.1038/s41377-025-01948-1

**Published:** 2025-09-15

**Authors:** Yigong Luan, Attilio Zilli, Agostino Di Francescantonio, Vincent Vinel, Paolo Biagioni, Lamberto Duò, Aristide Lemaître, Giuseppe Leo, Michele Celebrano, Marco Finazzi

**Affiliations:** 1 Department of Physics, Politecnico di Milano, 20133 Milan, Italy; 2 Université de Paris, CNRS, Laboratoire Matériaux et Phénomènes Quantiques, 75013 Paris, France; 3 Université Paris-Saclay, CNRS, Centre de Nanosciences et de Nanotechnologies, 91120 Palaiseau, France

**Keywords:** Nanophotonics and plasmonics, Nonlinear optics, Metamaterials

## Abstract

Optical metasurfaces allow complex light manipulation within subwavelength thicknesses and are thus rapidly emerging as a key enabling technology for nanophotonics applications. The control over light polarization already provided a route towards ultracompact metasurface-based polarimetry devices. If translated to the nonlinear optical regime it may become a transformative tool for nonlinear imaging, optical holography, and sensing. Here, we report ultrafast all-optical polarization modulation of upconverted light by all-dielectric metasurfaces via nonlinear interferometry. By controlling the relative phase between a pump beam at *ω* and its frequency-doubled replica at 2*ω*, we can set the phase relation between two frequency-degenerate upconversion processes at 3*ω* – sum-frequency generation and third-harmonic generation – stemming from an AlGaAs metasurface. By leveraging the opposite parity of the two nonlinear processes and adjusting their relative intensities, we achieve a modulation of the polarization state of the upconverted light between linear and circular states with a circular polarization degree of up to 83%. Remarkably, circularly polarized light of opposite handedness is symmetrically mapped in the Fourier space, at coincidence with the first diffraction orders of the metasurface. Furthermore, the handedness can be completely reversed within the same diffraction order by applying a phase delay of π. Our work adds an additional modulation layer beyond intensity to all-optical routing with precise phase control: polarization. The capability to encode and modulate simultaneously different polarization states in the *k*-space holds potential for chiral sensing and advanced imaging techniques.

## Abstract (web summary)

We demonstrate polarization encoding of upconverted light with orthogonal polarization states into opposite diffraction orders of a metasurface, enabling all-optical tuning between linear and circular polarization by adjusting the pump pulse phase delay.

## Graphical abstract

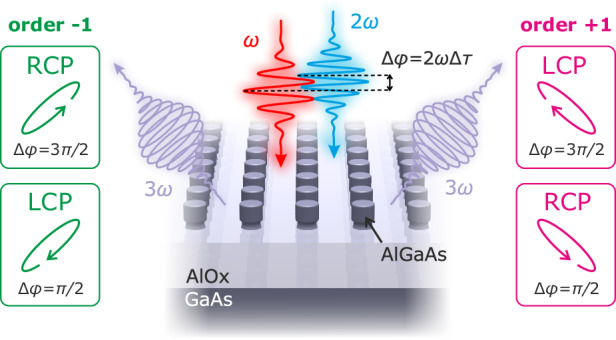

## Introduction

Metasurfaces—namely, engineered planar arrays of nanostructures (*meta-atoms*) of subwavelength thickness—are revolutionizing photonic technologies^1,2^, offering many new degrees of freedom for controlling light–matter interaction in confined volumes^3^. Beside their compactness compared to conventional bulk optical components, metasurfaces can be designed to feature multiple optical functionalities on a single platform, such as frequency upconversion^4,5^, wavefront shaping^6–8^, spectral filtering^9,10^, temporal control^11,12^, and quantum light generation^13,14^.

Polarization is a fundamental property of light that can be efficiently manipulated by metasurfaces^15,16^, making it possible to realize ultrathin versions of basic polarization optics components such as retarders and polarizers, as well as more complex devices such as polarimetric cameras for smart image recognition^17,18^. The adoption of nonlinear optical metamaterials^19–21^ offers also a unique alternative for manipulating light polarization. Indeed, the conservation law of angular momentum implies selection rules specific to each nonlinear process based on the crystal symmetry^22,23^, that apply as well at the mesoscopic scale of the meta-atom geometry^24,25^. Nonlinear optical activity is typically larger than its linear counterpart because of the high sensitivity of optical harmonics generated by circularly polarized light to asymmetry. For example, meta-atoms with discrete rotational symmetries (even if achiral) can produce harmonics polarized with opposite handedness to the pump^26,27^. Conversely, the generation of circularly polarized harmonics from a linearly polarized pump requires chiral meta-atoms^27^. Such design rules for polarization control have been recently exploited to realize all-optical switching^28^, polarization imaging^29,30^, and chiral sensing^31^ with nonlinear optical metasurfaces. All the examples listed above^26–31^ rely on complex geometries, with respect to either the shape or the arrangement of the meta-atom; this implies a significant design effort and has little tolerance to deviations from the design introduced by the nanofabrication. An alternative approach, termed *extrinsic chirality*, relies on the use of oblique incidence angles^32^. Yet, this comes at the expense of a more complex experimental arrangement implying either a tilted sample or a non-collinear detection.

In this work, we demonstrate an arrangement where the mirror symmetry is broken in detection instead of excitation, which we achieve by monitoring individually the off-axis diffraction orders of the nonlinear metasurface. This asymmetric detection enables us to encode simultaneously two orthogonal polarization states of the upconverted light in two specular diffraction orders, using linearly polarized pumps at normal incidence and a simple platform geometry consisting in a periodic array of AlGaAs nanocylinders. Specifically, the polarization of the upconverted light is ruled by the interplay between two frequency-degenerate nonlinear mixing processes, namely: third-harmonic generation (THG) at $3\omega =\omega +\omega +\omega$ and sum-frequency generation (SFG) at $3\omega =\omega +2\omega$. These processes are seeded by a pump pulse at (angular) frequency *ω* in the telecom C band (wavelength *λ* = 1556 nm) and its externally frequency-doubled replica at 2*ω*. We already reported such an interferometric, or coherent, control scheme in previous works based on plasmonic^33^ and dielectric^34^ nanostructures; however, these earlier demonstrations exploited the interference of co-polarized THG and SFG to achieve intensity modulation, whereas here the superposition of cross-polarized THG and SFG brings about polarization control.

In perspective, monitoring in parallel multiple diffraction orders makes a differential detection possible, hence potentially minimizing the common-mode source noise and mechanical fluctuations for better stability. Another key advantage peculiar to our approach is the ability to continuously tune the output polarization state, from linear to circular, by adjusting the relative phase between the two pumps. Such *reconfigurability*^35^ for a meta-device represents a highly sought-after feature for many applications, for instance in the rapidly evolving field of metasurface-based optical analog computing^2,36–39^. Many approaches to the dynamic reconfiguration of nonlinear optical signals have been proposed, including the use of liquid crystals^40,41^, as well as electro-optical^42,43^, thermo-optical^44^, and ultrafast mechanisms^45,46^. Among these, all-optical approaches^33,34,44–46^ are particularly promising, as they combine moderately high commutation efficiencies with contactless operation. Moreover, resorting to phase-based control, these approaches may reach extremely high commutation speed, enabling THz-speed reconfigurability^47^.

## Results and discussion

In our experimental realization, routing and polarization modulation of upconverted light is mediated by an all-dielectric AlGaAs metasurface. AlGaAs is a particularly advantageous material platform for photonic applications due to its high refractive index, which enables a strong confinement of the electromagnetic field, thus boosting light–matter interaction in nanostructures of subwavelength thickness. Its broad transparency window from the near- to the mid-infrared range of the electromagnetic spectrum makes it a versatile choice for a wide range of optical technologies. AlGaAs also features a high second-order nonlinear susceptibility (${d}_{36}$ > 100 pm/V), resulting in an efficient nonlinear conversion^48–52^.

**Fig. 1:**
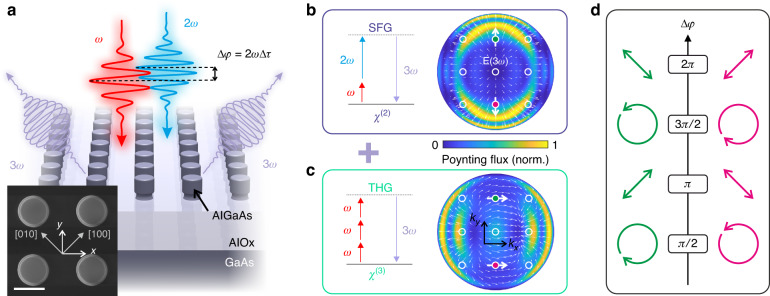
All-optical polarization modulation and routing. **a** Illustration of the nonlinear mixing between a pulse at frequency *ω* and its collinear frequency-doubled replica at 2*ω* mediated by an AlGaAs metasurface and the resulting polarization modulation at 2*ω*. The inset shows a scanning electron micrograph of the investigated metasurface highlighting the crystalline axes of AlGaAs and the chosen laboratory reference frame. Scale bar: 500 nm. Far-field angular distribution of the power emitted by an isolated meta-atom via (**b**) sum-frequency and (**c**) third-harmonic generation. The white arrow fields represents the electric fields at 3*ω*, with the large arrows highlighting their orientation in the (0, ±1) diffraction orders of the metasurface. **d** Schematics of polarization control as a function of the phase delay ∆φ between the *ω* and 2*ω* pump beams. Orthogonal polarization states are generated in the (0, +1) and (0, –1) diffraction orders (magenta and green colors, respectively, as in panels **b**, **c**), that are mirrored with respect to the origin of the *k*-space

Figure 1a presents the concept of polarization modulation via nonlinear interferometry: the investigated metasurface (details of the nanofabrication can be found in the Materials and Methods Section) enables the interaction between a fundamental femtosecond pulse at a frequency *ω* and its frequency-doubled replica at 2*ω*, allowing for a precise control over the phase difference of the upconverted fields at 3*ω* generated by either SFG or THG. The simulated (Comsol Multiphysics) far-field projections of the SFG or THG radiation by an isolated meta-atom, excited by horizontally (i.e. along *x*) co-polarized pump beams at *λ*_*ω*_ = 1550 nm and *λ*_2*ω*_ = 775 nm, are shown in panels b and c of Fig. 1. The electric field distributions feature orthogonal electric fields of the same magnitude for specific points in the *k*-space highlighted by colored circles (see Section S2 of the Supplementary Information and our previous work^34^ for the methods to retrieve the simulated back focal plane (BFP) maps). We tailor the periodicity *p* to 1000 nm to have the (0, ±1) diffraction orders of the metasurface overlapping with these points in the Fourier plane. We stress that at different input power levels other periodicity would fulfill the same criterion. In this way, by controlling the relative phase delay between the two pump beams at *ω* and 2*ω*, we gain control over the polarization states of the upconverted light in the two diffraction orders. The polarization at 3*ω* can be switched between circular and linear by varying the relative phase delay between SFG and THG (i.e., between the two pump beams). Note that the electric field (white arrows) is (anti)symmetric with respect to the *k*_*y*_ = 0 mirror plane for (SFG) THG. The opposite parity is due to the respectively even and odd number of input photons in SFG and THG, corresponding to their second (${\chi }^{(2)}$-mediated) and third (${\chi }^{(3)}$-mediated) nonlinear order. As a result, orthogonal polarization states (indicated in magenta and green in Fig. 1d) are simultaneously generated in specular points of the *k*-space with respect to the *k*_*y*_ = 0 plane. Finally, by toggling the relative phase delay between the two processes by *π*, the polarization state swaps between specular orders. We stress that, if we relied instead on the interplay of nonlinear processes of the same order (e.g. the same harmonic emission generated with orthogonal pumps) we would generate the same (arbitrary) polarization state in both specular orders for any given phase delay.

**Fig. 2:**
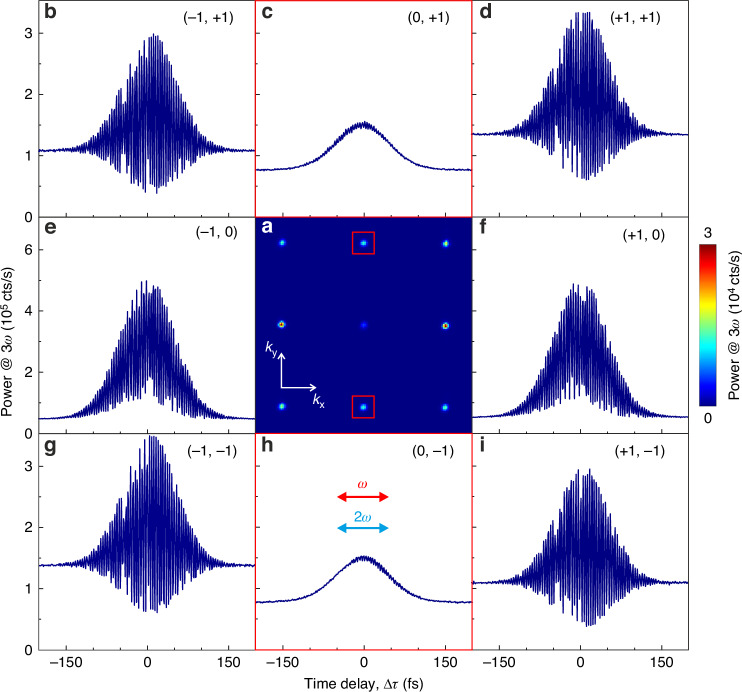
Nonlinear interferometry by an all-dielectric metasurface. **a** Experimentally delay-averaged BFP image showing the upconverted light power at 3*ω* (SFG + THG) from an AlGaAs metasurface with a lattice periodicity of 1000 nm. The image is produced by averaging 40 frames, with a relative delay between the pump pulses at *ω* and 2*ω* increasing in steps of 1 fs. The linear polarization of the pump beams is parallel to the *x* axis, with powers *P*_*ω*_ = 11 mW and *P*_2*ω*_ = 22 μW, respectively. **b**–**i** Delay traces of eight diffraction orders. The power diffracted at each phase delay is obtained by integrating a 13 × 13-pixel area around the center of the diffraction spots and subtracting the background

To put these numerical predictions to the test, we first recorded a delay trace longer than the pulse duration using a mechanical stage placed on the *ω* beam path and detected the intensity of the upconverted light (SFG + THG). Specifically, we performed the Fourier (*k*-space) imaging of the light upconverted by the metasurface by projecting the BFP of the objective onto a CCD camera through a 4 *f* telescope without introducing a polarization analyzer in the detection path (see Fig. 2). This allows identifying the diffraction orders and revealing where SFG and THG fields are orthogonal, which results in the absence of amplitude modulation upon changes of the relative phase. The polarization of the two pump beams is horizontal in the laboratory reference (*x* axis) as in the simulations of Fig. 1b, c. A detailed description of the experimental setup is presented in the Materials and Methods section and Section S3 of the Supplementary Information. Figure 2a presents the delay-averaged BFP image of the upconverted light at 3*ω*. This image is obtained by averaging 40 frames captured at 1 fs delay intervals around the arbitrary zero-delay condition. The diffraction orders (0, +1), (0, –1), (–1, 0) and (+1, 0) are anticipated at a numerical aperture (NA) of 0.52, computed as ${\rm{NA}}={\lambda }_{3\omega }/p$. Figure 2b–i is the delay traces of all the diffraction orders within the collection NA (0.85) spanning the range from –200 fs to +200 fs, acquired with a delay resolution of 1 fs. The power of each diffraction order is measured by averaging a 13 × 13 pixel^2^ area centered on the most intense pixel and applying a local background subtraction procedure (see Section S7 of the Supplementary Information). Each delay trace displays a baseline of THG when the delay between the *ω* and 2*ω* pulses is much longer than their duration, while SFG occurs on the top of THG at shorter delays, with the maximum of the envelope identifying the zero-delay condition. All diffraction orders except (0, ±1) in panels c and h exhibit prominent interference fringes. This is in line with our recent findings^34^, and is attributed to the modulation of the relative phase between SFG and THG. In contrast, Fig. 2c, h (red squares) display negligible interference in the (0, ±1) diffraction orders, pointing towards orthogonal field polarizations of SFG and THG. These two diffraction orders are thus interesting for achieving tunable polarization states through phase modulation.

**Fig. 3:**
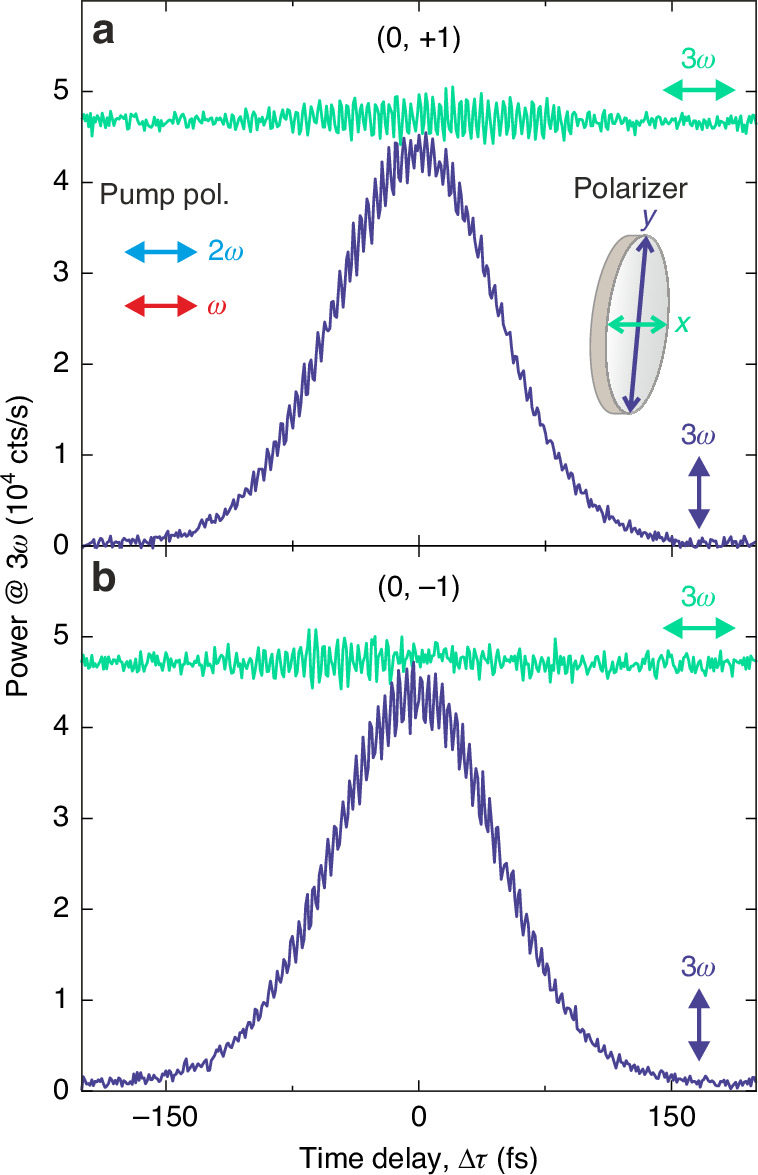
Disentangling THG and SFG signals by polarized detection. **a** Delay traces of the (0, +1) diffraction order at 3*ω*, analyzed with a linear polarizer selecting the light linearly polarized along the *x* (green) or *y* (blue) axis in the detection path. When the linear polarizer is aligned along *x*, the trace shows a delay-independent behavior, ascribed to the THG component (green). In contrast, when the linear polarizer is aligned along *y*, the trace exhibits a bell-shaped profile with null offset (blue), characteristic of the SFG component. THG and SFG powers at the zero-delay condition have been equalized by adjusting the pump powers, to enable polarization modulation between orthogonal circular polarization states. **b** Same as (**a**) for the (0, –1) diffraction order

To validate this evidence, we analyzed the polarization of the light emitted at 3*ω* into these orders by inserting a linear polarizer in the detection path. This allows us to disentangle the contribution of the upconverted signals polarized along the *x* or the *y* axis and, hence, to verify if the two frequency-degenerate signals do indeed possess orthogonal linear polarizations. Figure 3a, b shows the delay traces with polarization selection along the *x* (green traces) and the *y* axis (blue traces) for the (0, ±1) diffraction orders exhibiting negligible interference in Fig. 2c, h. The powers diffracted in both diffraction orders are delay-independent when the polarizer is aligned along *x*, which is characteristic of THG seeded by the *ω* beam. Concurrently, the delay traces show a bell-shaped profile when the analyzer is aligned along *y*, indicating an upconversion process produced by the temporal superposition of the beams *ω* and 2*ω*, which we therefore identify as SFG. The small residual fringes arise from deviations from a perfectly orthogonal alignment between the polarizations of the SFG and THG fields. The THG and SFG powers at zero-delay have been equalized to obtain circular polarization states rather than just elliptical. This balancing has been achieved by adjusting the impinging powers of the *ω* and 2*ω* pump beams to *P*_*ω*_ = 11 mW and *P*_2*ω*_ = 22 μW. Such pump power equalization, also employed to retrieve the results in Figure 2, is crucial to maximize the polarization modulation in these diffraction orders, as shown below. We highlight that by rotating the polarization of the 2*ω* beam by 90°, that is, by toggling the polarization configuration of the pumps from co-polarized to cross-polarized, such a configuration of the electric fields is routed from the (0, ±1) diffraction orders to the (± 1, 0) ones. The experimental data collected with cross-polarized pumps can be found in Section S4 of the Supplementary Information, including the same measurements shown in Figs. 2, 3 for the co-polarized configuration. This polarization-induced reconfiguration demonstrates the flexibility of our optical control scheme, which allows routing the polarization-modulated diffraction orders to different sets of directions, namely along either *k*_*x*_ or *k*_*y*_.

**Fig. 4:**
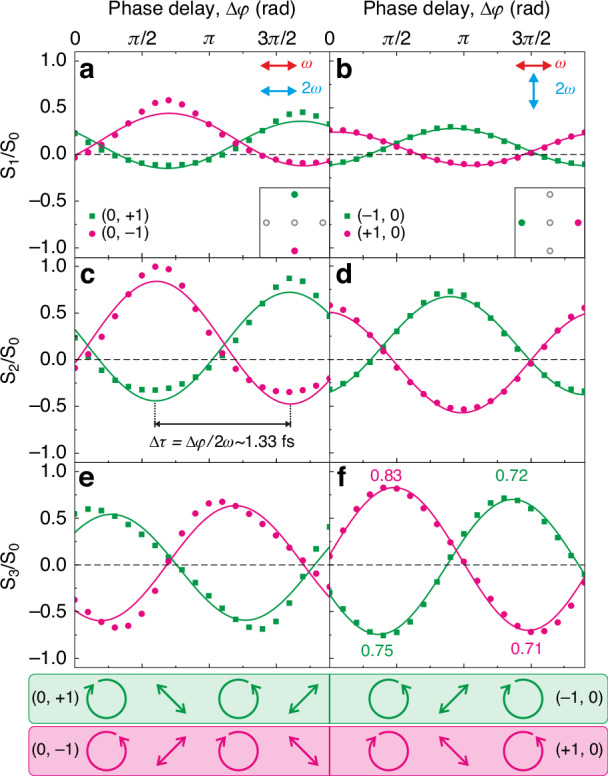
Normalized Stokes parameters as functions of relative phase delay. Normalized Stokes parameter *S*_1_*/S*_0_ for (**a**) the (0, +1) (squares) and (0, –1) (dots) diffraction orders when co-polarized pump beams aligned along the *x* axis are employed and (**b**) the (–1, 0) (squares) and (+ 1, 0) (dots) diffraction orders when cross-polarized pump beams are employed. *S*_1_*/S*_0_ shows the residual vertical and horizontal polarization states in the modulation. The solid curves are sinusoidal fits. Inset in a and b: representation of the diffraction orders investigated. Normalized Stokes parameter (**c**, **d**) *S*_2_*/S*_0_, and (**e**, **f**) *S*_3_*/S*_0_ for the same diffraction orders as in (**a**) and (**b**). The magenta and green bars help identifying the modulation between linear and circular polarization states of the respective orders in panels c–f. Numbers in panel (**f**) are the maximum DOCP attained

Finally, to demonstrate the continuous polarization modulation, that is, to achieve a fine tuning of the 3*ω* polarization state, we introduce a half-wave compensated liquid-crystal retarder in the *ω* beam arm of the interferometer. By varying the applied voltage between 0 and 10 V, we induce a relative phase delay ($\Delta \varphi =2\omega \cdot \Delta \tau$) ranging from 0 to 2*π* with a step-size of 0.1*π*, corresponding to a time delay of 150 as. To fully characterize the polarization of the upconverted light at 3*ω*, we employed the rotating quarter-waveplate polarimetry, as described by Schaefer et al.^53^ and Section S5 of the Supplementary Information. Experimentally, this requires inserting a rotating quarter-waveplate in the detection path followed by a fixed linear polarizer (here aligned with its transmission axis aligned along *x*). The normalized Stokes parameters are then evaluated over a full optical cycle based on the diffracted powers from the BFP images. Figure 4 shows the normalized Stokes parameters as a function of the relative phase delay for two different pump polarization configurations, namely, co-polarized (left column) and cross-polarized (right column). The normalized Stokes parameters *S*_1_/*S*_0_ and *S*_2_/*S*_0_ describe the degree of linear polarization (DOLP), corresponding to the intensity differences between orthogonal polarization states along *x*/*y* (0°/90°) and diagonal ( + 45°/ − 45°) axes, respectively. In contrast, *S*_3_*/S*_0_ characterizes the degree of circular polarization (DOCP), reflecting the balance between right- and left-handed circularly polarized light. By observing the sinusoidal behavior (see the solid curves) of the (0, +1) diffraction order one can notice that *S*_1_*/S*_0_ and *S*_2_*/S*_0_ oscillate in phase, while *S*_3_*/S*_0_ has a *π*/2 phase delay. This indicates a periodic transition between linear and circular polarization states as the relative phase delay between the two pump beams changes. The deviation of the experimental data from the sinusoidal fits can be attributed to phase fluctuations caused by mechanical drifts of the interferometer arms. Furthermore, because of the opposite parity of the two nonlinear fields involved, the normalized Stokes parameters of the specular (0, ±1) diffraction orders are in antiphase. As a result, circular polarized light with opposite handedness and high DOCP is generated in specular orders for a specific relative phase delay. Notably, the handedness of the circular polarization within the two orders can be completely reversed by introducing an additional *π* phase delay. The modulation mechanisms ascribed to *S*_3_*/S*_0_ are schematized for clarity in the colored bars below panels e and f.

Among all the configurations, we report a maximum value for the DOCP of 83%. We stress that this value, which theoretically can reach up to 100%, is only limited by two factors. The first limitation resides in the linear dichroism introduced by the optical elements in the detection path, as it was confirmed by measuring the DOCP of a circularly polarized laser (*λ* = 520 nm) that got reduced to 85% (see section S6 in Supplementary Information). This clearly indicates an underestimation of the DOCP generated by the metasurface. A second minor limitation, which directly affects the DOCP generated by the metasurface, is an imperfect balancing in the relative powers of SFG and THG fields as well as a non-perfectly orthogonal polarization state. Both factors lead to elliptical polarization states and a non-vanishing value of the *S*_1_*/S*_0_ ratio (see Fig. 4a, b) as well as a reduced amplitude of the *S*_2_*/S*_0_ and *S*_3_*/S*_0_ ratios (see Fig. 4c–f).

## Conclusions

We demonstrated the upconversion of telecom photons (frequency *ω*) to the visible range by frequency tripling at 3*ω* using an AlGaAs metasurface, and the interferometric control of the polarization state of the upconverted light. This is achieved by the interplay between SFG and THG processes, coherent and degenerate in frequency at 3*ω*, that are seeded by a dual-beam (*ω* + 2*ω*) pumping scheme. The metasurface was engineered to feature specific diffraction orders, where SFG and THG have equal power and orthogonal linear polarization, so that the polarization state of the nonlinear emission can be tuned continuously from linear to circular within the same order, by adjusting the relative phase delay between the two pump beams. A degree of circular polarization larger than 80% was achieved, mainly limited by the linear dichroism of the optical elements in the detection path. Notably, by monitoring individually off-axis diffraction orders, the mirror symmetry of the system is broken, which allows us to generate circular polarization states from a linearly polarized input light without resorting to chiral nanostructures^27^ nor to non-normal incidence on the sample^32^ as in established approaches. Moreover, nonlinear processes of opposite parity (SFG and THG are examples of a ${\chi }^{(2)}$ and a ${\chi }^{(3)}$ process, respectively) can produce two orthogonal polarization states simultaneously—e.g., opposite handedness—in specular diffraction orders. Finally, the set of specular diffraction orders which are modulated in polarization can be re-routed into an orthogonal set—that is, from (0, ±1) to (±1, 0)—by switching the pump polarization from co- to cross-polarized, affording additional flexibility. Since it does not rely on specific resonant behavior nor material system, our concept is quite robust and easily adaptable to different platforms and, in principle, even to different nonlinear processes. It is indeed always possible to sample different points in *k* space by changing the pitch of the metasurface, and to adjust the relative power and polarization of the pumps to bring balanced, cross-polarized nonlinear emission to interfere. In perspective, the capability to encode with a high modulation speed on-demand polarization states into different channels (i.e. points of the Fourier space), which can be continuously adjusted from linear to circular, discloses great potential in chiral sensing. Specifically, the balanced detection of the two specular orders would provide a differential signal to sense the adhesion of chiral molecules to the sample. Unlike conventional methods that require modulating the relative phase delay between orthogonal polarization components to switch between orthogonal polarizations states, our approach eliminates the need to actively modify the relative phase delay. Instead, orthogonal polarizations are generated simultaneously at different positions in the BFP. Differential detection of the two oppositely polarized diffraction orders will thus be less sensitive to the intensity fluctuations of the laser source than phase-modulation polarimetry, enabling low-noise enantiosensitive characterizations^54^. Moreover, the ability to control in parallel the polarization states of distinct diffraction orders can extend the potential of this approach even to quantum computing and advanced imaging techniques, further broadening its impact.

## Materials and methods

### Sample fabrication

The metasurface is a periodic (*p* = 1000 nm) array of Al_0.18_Ga_0.82_As nanocylinders, each 400 nm in height and 500 nm in diameter, oriented along the [110] and [$1\bar{1}0$] AlGaAs crystal axes. These nanocylinders are fabricated via electron-beam lithography and supported by a low-refractive index AlOx substrate (*n* = 1.6), resulting in a strong field confinement within the AlGaAs nanocylinders (*n* = 3.2)^49^. The 18% Al doping which is employed to prevent modification of the nanocylinders during the realization of the AlOx substrate by annealing^49^, also imparts a shift to the material bandgap to a wavelength shorter than 750 nm, which suppresses two-photon absorption to further enhance the nonlinear conversion efficiency^52^. In this configuration, each nanocylinder supports an electric dipole resonance at a pump wavelength of 1556 nm^34^. A detailed description of the fabrication is included in Section S1 of the Supplementary Information and in Gili et al.^49^.

### Experimental setup

The laser source (NKT Photonics, Origami 15 LP) generates pulses with a duration of 200 fs and a repetition rate of 80 MHz, centered at an angular frequency *ω*, corresponding to a wavelength of 1556 nm. The beam is partially converted to 2*ω* with a β barium borate crystal (Eksma Optics, βBBO-SHG@1554 nm), producing a wavelength of 778 nm. The two pulses are separated through a short-pass dichroic mirror (Thorlabs, DMSP950), with the relative temporal delay controlled by a mechanical delay stage (Physik Instrumente, M-404) in the *ω* beam path, allowing for adjustments with a minimum step of about 1 fs. Subsequently, the *ω* and 2*ω* beams are recombined with another short-pass dichroic mirror (Thorlabs, DMSP950) and focused onto the BFP of an air objective (Nikon, CFI Plan Fluor 60XC, NA = 0.85) using an achromatic lens doublet (Thorlabs, AC254-500). This configuration enables a nearly collimated illumination on the sample, resulting in a spot size of about 20 *μ*m. Half-wave retarders (Thorlabs, WPH05M-1550 and WPH05M-808) are inserted in the *ω* and 2*ω* beam paths, respectively, to independently adjust the polarization of the two beams. The nonlinear emission at 3*ω* is collected by the same objective in a back-scattering configuration and separated from the excitation by a long-pass dichroic mirror (Thorlabs, DMLP650). The emission is then filtered by spectral filters (Thorlabs, FESH1000 + FESH700 + FBH520-40) to select the wavelength centered around 518 nm (3*ω*). Eventually, the back focal image of the objective at each phase delay is relayed onto a silicon CCD camera (Andor, iKon-M DU934P-BV) with a 4 *f* imaging system composed of a pair of achromatic doublets (Thorlabs, AC508-500-B). As an analyzer for the linear polarization detection experiment (see Fig. 3) we inserted a linear polarizer (Thorlabs, LPVISB100-MP2) in the detection path. High resolution phase delay plots were obtained by inserting a half-wave compensated liquid-crystal retarder (Thorlabs, LCC1411-C) centered at 1551 nm with its slow axis aligned parallel to the polarization of the *ω* beam. The resolution in the phase delay is about 0.1*π*, which is equivalent to a temporal delay of 150 attoseconds^34^. For the rotating quarter-waveplate polarimetry a rotating achromatic quarter-waveplate (Thorlabs, AQWP10M-580) is followed by a fixed linear polarizer (Thorlabs, LPVISB100-MP2) with its transmission axis aligned along the *x*-axis. The detailed procedure of the evaluation of the Stokes parameters is included in Section S5 of the Supplementary Information.

## Supplementary information

Supplementary Information

## Data Availability

All data that support the findings of the study are provided in this article and the Supplementary Information file. The raw data generated in this study have been deposited in the Zenodo database with DOI:10.5281/zenodo.15855413.
