# Supplementary material for: All-optical polarization encoding and modulation by nonlinear interferometry at the nanoscale
Source: Light Sci Appl. 2025 Sep 15;14:318. doi: 10.1038/s41377-025-01948-1 (PMC12434142; doi:10.1038/s41377-025-01948-1)
Supplement: Supplementary file 1 — Supplementary Information [file 41377_2025_1948_MOESM1_ESM.pdf]

Supplementary information for:

All-optical polarization control and routing by nonlinear interferometry at the nanoscale

Yigong Luan,[1] Attilio Zilli,[1] Agostino Di Francescantonio,[1] Vincent Vinel,[2] Paolo Biagioni,[1] Lamberto Duò,[1] Aristide Lemaître,[3] Giuseppe Leo,[2] Michele Celebrano,[1] Marco Finazzi[1*]*

1.Department of Physics, Politecnico di Milano, Milano 20133, Italy

2.Université de Paris, CNRS, Laboratoire Matériaux et Phénomènes Quantiques, Paris 75013, France

3.Centre de Nanosciences et de Nanotechnologies, CNRS, Université Paris-Saclay, Palaiseau 91120, France

[*]Correspondence to: michele.celebrano@polimi.it; marco.finazzi@polimi.it

Outline:

S1: Fabrication process of metasurfaces ***2***

S2: Simulation of back-focal plane image ***3***

S3: Experimental setup ***5***

S4: Delay traces with xy polarization ***6***

S5: Evaluation of the Stokes parameters ***8***

S6: Characterization of polarization dichroism of the collection optics ***11***

S7: Conversion efficiency and coefficient of SFG and THG ***13***

S1: Fabrication process of metasurfaces

The dielectric metasurface studied in this work is fabricated via a lithographic process on the epitaxial structure shown in Fig.S1(a) and consisting in a stack encompassing (i) a bulk GaAs wafer, (ii) a 90 nm $Al_xGa_{1-x}As$ transition layer with increasing aluminium concentration, (iii) a 1.5 µm-thick AlOx layer, (iv) a second 90 nm $Al_xGa_{1-x}As$ transition layer, and (v) a 400 nm-thick $Al_{0.18}Ga_{0.82}As$ layer that is subsequently nanostructured. The latter is deposited on top of the aluminium-rich substrate by molecular beam epitaxy (MBE) (panel b). A Hydrogen silesquioxane negative resist is then spin-coated on the sample (panel c) after a thin 10 nm-thick SiO_2 film acting as adhesion promoter

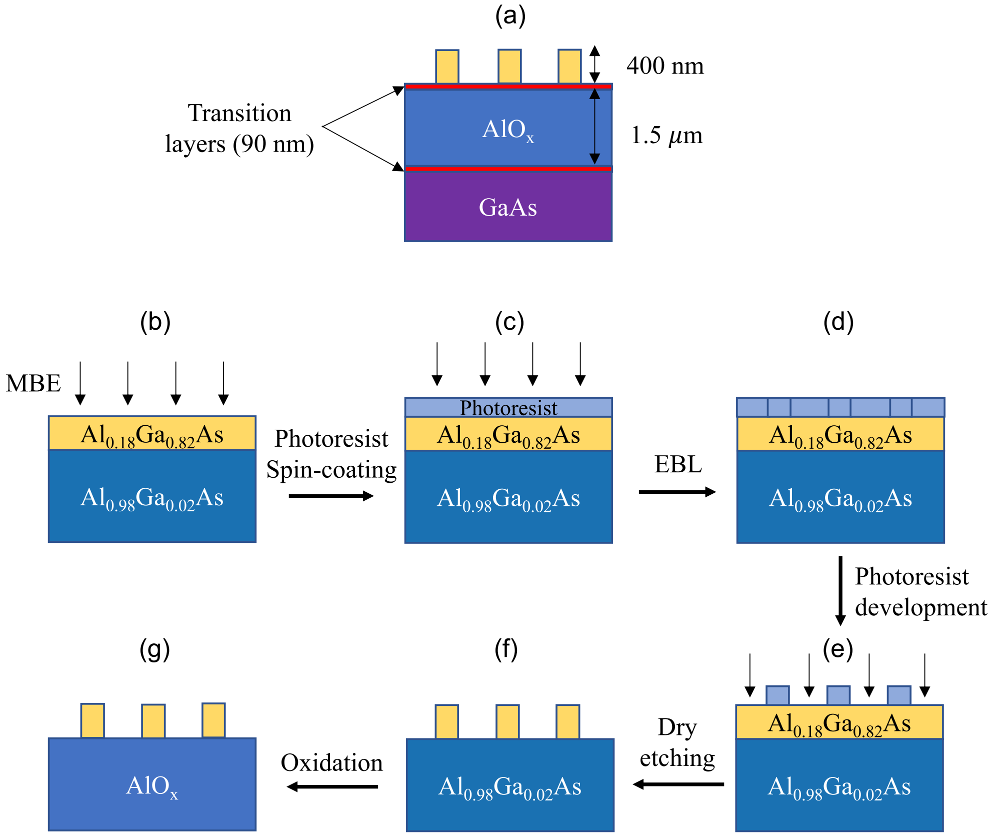

Figure S1: The fabrication process of AlGaAs metasurfaces. (a) Sketch of the epitaxial structure of the AlGaAs sample. (b) Deposition of an $Al_{0.18}Ga_{0.82}As$ layer on aluminum-rich AlGaAs by MBE. (c) Spin-coating of the photoresist. (d) electron-beam lithography (EBL). (e) Development of the photoresist. (f) Inductively coupled plasma reactive ion etching (ICP-RIE). (g) Oxidization of the substrate.

is realized by plasma-enhanced chemical vapor deposition (PECVD). Subsequently, arrays of equally spaced nanopillars are patterned employing 20 kV electron-beam lithography (panel d). After resist development (AZ 400K), the SiO_2 layer is removed by reactive ion etching (RIE) in CHF_3 gas (panel e). The lithographic pattern is subsequently transferred onto the $Al_{0.18}Ga_{0.82}As$ layer via inductively coupled plasma reactive ion etching (ICP-RIE) with $SiCl_4$ gas (panel f). Finally, the $Al_{0.98}Ga_{0.02}As$ layer was selectively oxidized to form AlOx at 390 °C for 40 minutes (panel g).

S2: Simulation of back-focal plane image

The back focal plane (BFP) images of the collected third-harmonic generation (THG) and sum-frequency generation (SFG) light are simulated in the frequency domain using the finite-element method (FEM) and the COMSOL Multiphysics solver. The model includes a periodic structure with a unit cell comprising a single AlGaAs (n = 3.2) cylinder (height h = 400 nm, radius r = 250 nm) placed on an AlOx layer (thickness t_{sub} = 1530 nm, refraction index n_2 = 1.6) and followed by a semi-infinite GaAs layer (dispersion relation from literature[1]). To account for the near-field coupling, Floquet periodicity along the x and y axes is incorporated with a period p = 1000 nm. A perfectly matched layer is used at the top and bottom boundaries to simulate infinite media. First, the local electric-field distribution at frequencies ω and 2ω is calculated in a scattered-field formulation with an analytical background field corresponding to a plane wave transmitted through a three-layer medium.[2] Then, the local electric field at frequency 3ω is calculated in a full-field formulation with the source given by the nonlinear polarization P_{SFG} and P_{THG} in the cylinder:

$$P_{i,\mathrm{SFG}}(3\omega) = 2\epsilon_0\chi^{(2)}_{ijk}\left[E_j(\omega)E_k(2\omega) + E_j(2\omega)E_k(\omega)\right],$$

$$P_{i,\mathrm{THG}}(3\omega) = \epsilon_0\left[\chi^{(3)}_{iiii}E_i^3(\omega) + 3\chi^{(3)}_{iijj}E_i(\omega)E_j^2(\omega) + 3\chi^{(3)}_{iikk}E_i(\omega)E_k^2(\omega)\right],$$

Here the indices $i \neq j \neq k$ identify the crystalline axes. $\mathbf{E}(\omega)$ and $\mathbf{E}(2\omega)$ are the local

electric fields calculated within the scattered-field formulation. In this simulation we use the values of $\chi^{(2)} = 2 \times 10^{-10}$ mV^{-1} and $\chi^{(3)} = 7 \times 10^{-20}$ m^{2}V^{-2} reported in another work.[3] The calculated local electric field at 3ω is then projected to the far-field with the RETOP toolbox.[4] Near-field coupling between the cylinders is neglected, since the period is much larger than the emitted wavelength of 518 nm. This method allows us to estimate the far-field projection of free-space radiation $\mathbf{E}_{\mathrm{THG}}(\theta, \varphi)$ and $\mathbf{E}_{\mathrm{SFG}}(\theta, \varphi)$. Subsequently, the angular power density of the THG and SFG emission in the Fourier plane is computed as $|S_{\mathrm{BFP}}(k_x, k_y)| = \frac{|S_{\mathrm{BFP}}(\theta, \varphi)|}{n^2 * \cos(\theta)}$, where **S** is the time-averaged Poynting vector and the cosine factor is the Jacobian of the transformation corresponding to the objective lens, modelled as an ideal aplanatic system.[2] Finally, the far-field emission from the metasurface (diffraction orders) is evaluated by multiplying $\mathbf{S}_{\mathrm{THG}}(k_x, k_y)$ or $\mathbf{S}_{\mathrm{SFG}}(k_x, k_y)$ with the array factor $\mathrm{A}(k_x, k_y)$ of the metasurface:

$$E_{\mathrm{THG,SFG}}^{\mathrm{Array}}(k_x, k_y) = E_{\mathrm{THG,SFG}}(k_x, k_y) \times A(k_x, k_y),$$

$$A(k_x, k_y) \propto \left(\frac{1 - e^{iMpk_x}}{1 - e^{ipk_x}}\right) \times \left(\frac{1 - e^{iNpk_y}}{1 - e^{ipk_y}}\right),$$

$M = N = 15$ (corresponding to 225 nonlinear emitters) agrees with the estimated size of the pump spots on the sample (about 20 μm) and reproduces well the experimental full-width-at-half-maximum size of the diffraction orders. The near-field distribution at the two excitation wavelengths 1550 nm and 775 nm are displayed in Fig.S2.

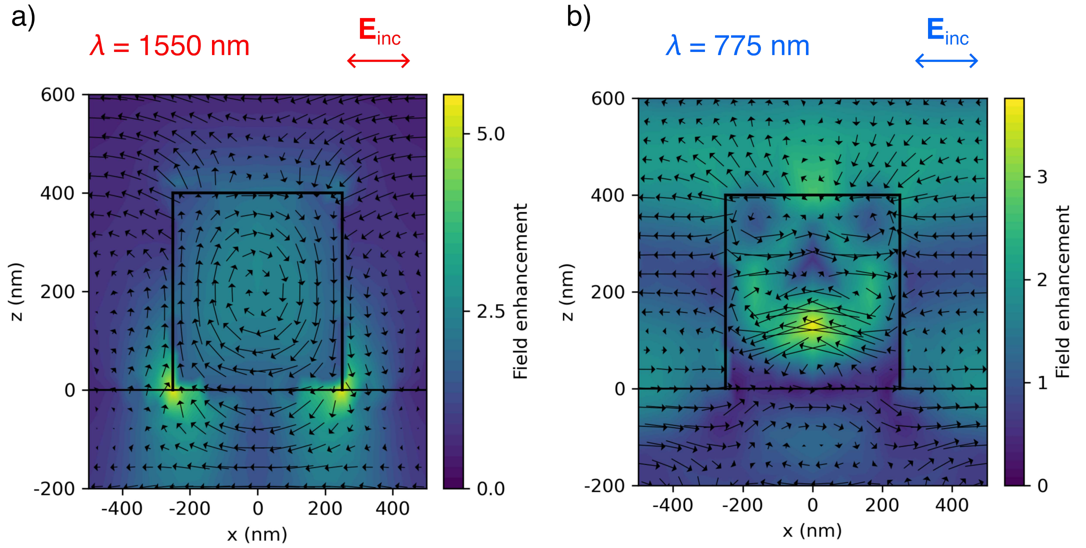

Figure S2: Near-field distribution $|\mathbf{E}|/|\mathbf{E}_0|$ at the pump wavelength 1550 nm (a) and 775 nm (b) for the investigated system with 1000 nm periodicity.

S3: Experimental setup

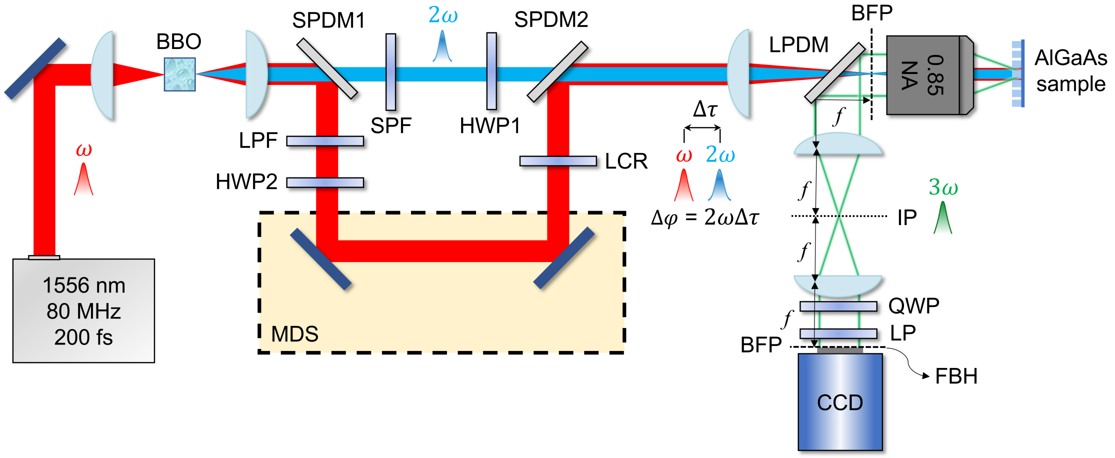

Figure S3: Diagram of the experimental setup. Acronyms: BBO: beta barium borate crystal; SPDM: short-pass dichroic mirror; LPF: long-pass filter; SPF: short-pass filter; HWP: half-wave plate; MDS: mechanical delay stage; LCR: liquid-crystal retarder; LPDM: long-pass dichroic mirror; BFP: back focal plane; IP: image plane; QWP: quarter-wave plate; LP: linear polarizer; FBH: hard-coated bandpass filter; CCD: charge-coupled device camera.

S4: Delay traces with *xy* polarization

As shown in Fig.2 of the article, under xx polarization of the two pump beams, the polarization state can be modulated at the (0, +1) and (0, -1) diffraction orders. This modulation arises from the superposition of cross-polarized THG and SFG, which exhibit equal intensities, as confirmed by the polarization-resolved delay traces in the paper (Fig.3). Here, Fig.S4 and Fig.S5 present the unpolarized and polarized delay traces for xy polarization of the two pump beams with powers $P_{\omega} = 11$ mW and $P_{2\omega} = 37$ μW, respectively. In this case, the polarization can be modulated at the (-1, 0) and (+1, 0) diffraction orders. The intensity modulation observed in Fig.S5 results from imperfections in the alignment of the two pump beams and their polarizations. These fringes, which are about one order of magnitude weaker than the overall signal, are ascribed to residual co-polarized field components of the SFG and THG. Additionally, spurious polarization components could also be introduced by the excitation and detection via a high-NA objective.

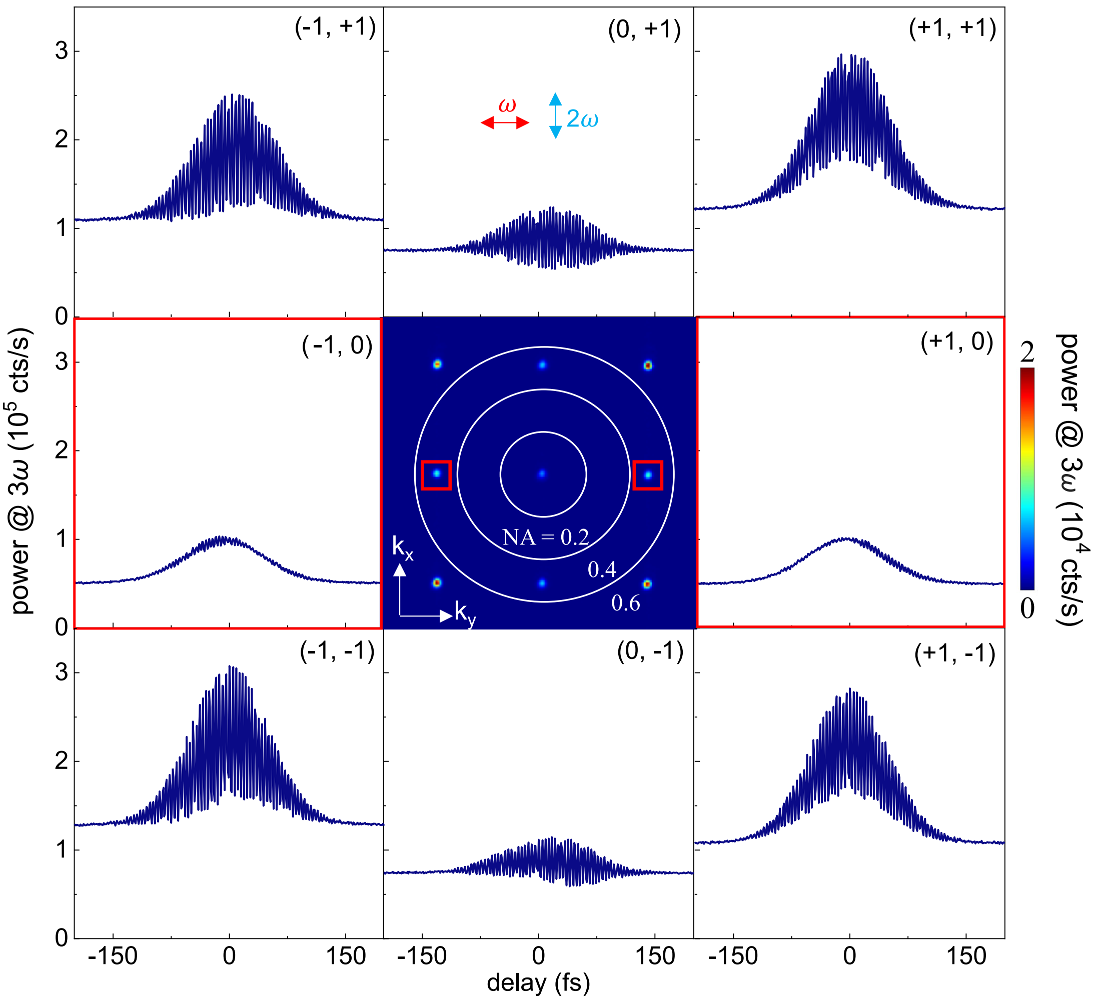

Figure S4: Central panel: Experimentally delay-averaged BFP image for the metasurface discussed in the main text (period p = 1000 nm) under xy polarization for ω and 2ω beams with powers of P_{ω} = 11 mW and $P_{2\omega}$ = 37 μW on the sample. The image is produced by averaging 40 frames, with a relative delay of 1 fs between two pump pulses. The power of diffraction spots at each phase delay is obtained by integrating a 13×13 pixels area near the center of the diffraction spot and subtracting the background. External panels: Delay traces of the first eight lowest diffraction orders, showing either interference fringes or no interference, because of the relative SFG and THG polarizations.

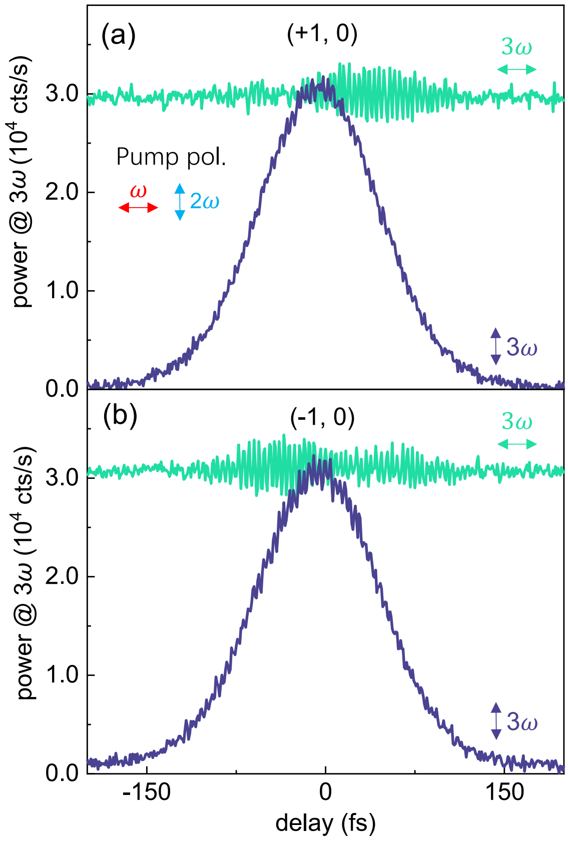

Figure S5: (a) Delay traces of the (+1, 0) diffraction order power, measured with a linear polarizer in the detection path selecting x- (green) or y-polarized (blue) light. (b) Same as (a) for the (0, -1) diffraction order.

S5: Evaluation of the Stokes parameters

The Stokes parameters of the upconverted light have been evaluated by positioning a quarter-waveplate rotated by an angle θ followed by a linear polarizer with its transmission axis aligned along the x-axis. In this configuration, the intensity of the transmitted optical beam is given by:[5]

$$I(\theta) = \frac{1}{2}(S_0 + S_1 \cos^2 2\theta + S_2 \cos 2\theta \sin 2\theta + S_3 \sin 2\theta),$$

S_0, S_1, S_2 and S_3 represent the four Stokes parameters for a plane wave. S_0 describes the total intensity of the optical field, S_1 quantifies the amount of linear horizontal or vertical polarization, S_2 quantifies the amount of linear +45° or -45° polarization, and S_3 is associated with the amount of right or left circular polarization. The Stokes parameters can be estimated by measuring the transmitted intensity I as a function of θ:[6]

$$S_0 = \frac{2}{N}\sum_{n=1}^{N} I(\theta_n) - \frac{4}{N}\sum_{n=1}^{N} I(\theta_n)\cos 4\theta_n,$$

$$S_1 = \frac{8}{N}\sum_{n=1}^{N} I(\theta_n)\cos 4\theta_n,$$

$$S_2 = \frac{8}{N}\sum_{n=1}^{N} I(\theta_n)\sin 4\theta_n,$$

$$S_3 = \frac{4}{N}\sum_{n=1}^{N} I(\theta_n)\sin 2\theta_n,$$

where N is the number of data points collected at different angles (see Fig.S6a).

The power of each diffraction order at 3ω is evaluated as a function of the angle θ and the relative phase delay $\Delta\varphi$ by integrating a 13×13 pixel^2 area around each order after background subtraction. Fig.S6a displays the power of the single polarization-modulated (-1, 0) diffraction order excited with xy polarization of the two pump beams (see Section S4). The color map shows the powers recorded at each rotation angle θ of the quarter waveplate as a function of delay (see in Fig.S6(a)). To evaluate the Stokes parameters, sinusoidal fits are first applied to all the delay scans at each fixed θ value, to compensate phase fluctuations introduced by the setup. The smoothed data are shown in Fig.S6(b). Subsequently, the interpolated intensities at each phase delay are extracted from the fitted curves and then used to calculate the Stokes parameters through Equation 2. Fig.S6(c) shows one set of raw data when the relative phase delay between two pump beams is 0.5π in comparison to the sinusoidal fitted data.

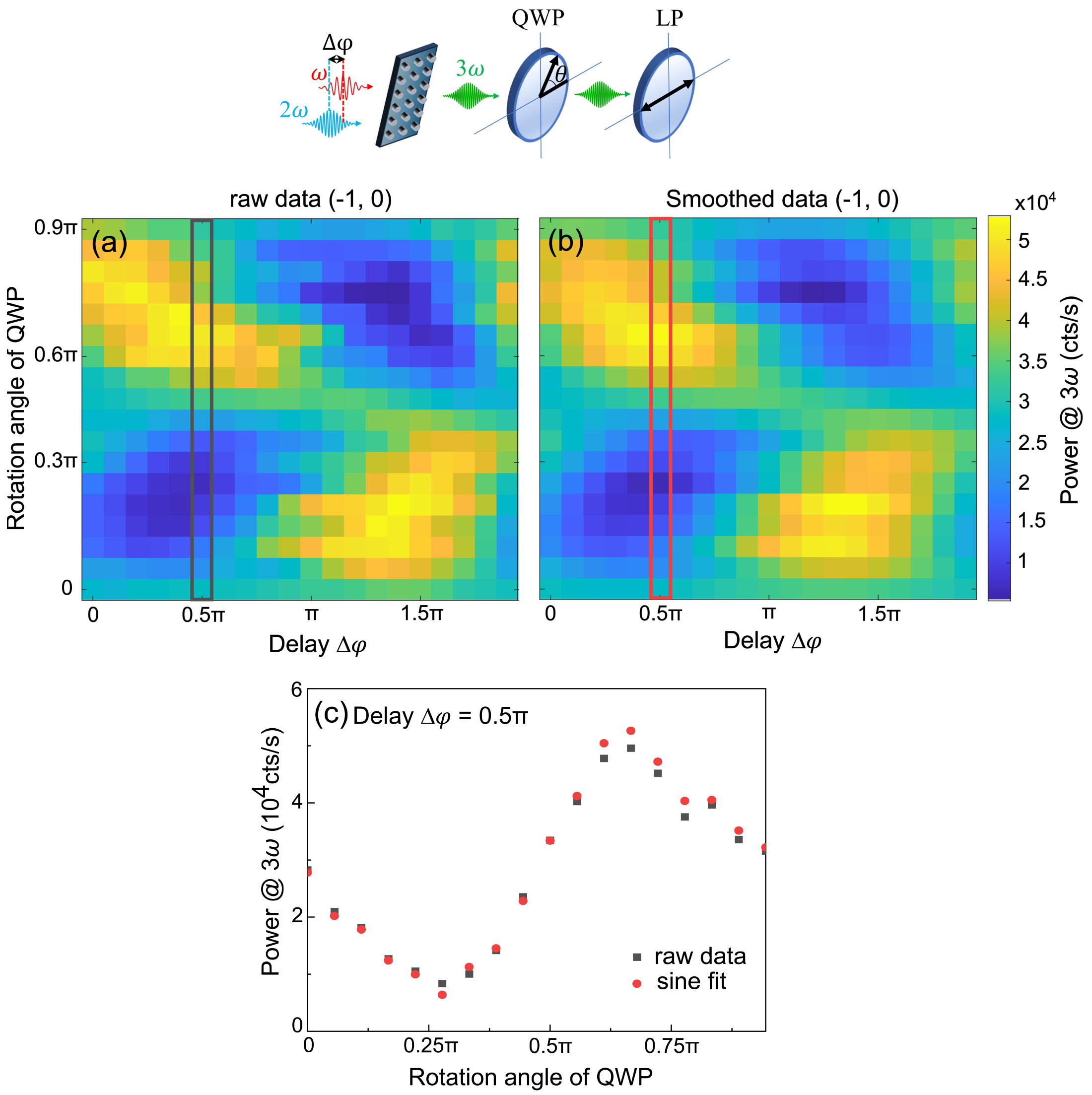

Figure S6: Upper panel: Simplified diagram of the polarimetry setup (QWP: quarter waveplate; LP: linear polarizer). (a) Power of diffraction order (-1, 0) at each rotation angle of the quarter-waveplate (y axis) as a function of delay (x axis). (b) Smoothed data after fitting with sinusoidal functions to each dataset obtained at fixed θ values. (c) One example showing the comparison between experimental raw data and sinusoidal fitted data when delay is at 0.5π.

S6: Characterization of polarization dichroism of the collection optics

To investigate the factors responsible for the reduction of the degree of circular polarization (DOCP) from 100%, we conducted a test consisting in sending the circularly polarized emission (either left- or right-handed) of green laser source (518 nm) through the detection path to assess the possible presence of dichroism and/or birefringence effects introduced by the optical elements.

As illustrated in Fig.S7(a), the green laser (Thorlabs, PL201) light was first linearly polarized along the y-axis using a Glan–Taylor polarizer (Thorlabs, SM1PM10) and then loosely focused at the sample position in front of the collection objective (Nikon, CFI Plan Fluor 60XC, NA = 0.85) to mimic the zero-order emission of the sample at 3ω. To generate circular polarization states, an additional quarter-wave plate (B. Halle, 500–900 nm achromatic) was placed before the focusing lens. The circularly polarized light propagated through the same optical components as the upconverted light in the experiment. In detail, the beam was reflected by a long-pass dichroic mirror (Thorlabs, DMLP650) and directed into the polarimetry setup, which included a quarter-wave plate (Thorlabs, AQWP10M-580) and a linear polarizer (Thorlabs, LPVISB100-MP2). Finally, the laser power was measured using a silicon power meter (Thorlabs, S120C).

Fig.S7(b) shows the recorded power for left- and right-handed circularly polarized light when the quarter-wave plate was positioned before the focusing lens (45°: left-handed, black; 135°: right-handed, red). The retrieved DOCP values were 87.5% (left-handed) and 83.2% (right-handed), closely matching the highest DOCP observed in the polarization modulation experiment (83.3%).

To determine whether this deviation from 100% DOCP was due to optical elements in the detection path, we repositioned the quarter-wave plate after the dichroic mirror. The recorded power values, shown in Fig.S7(c), confirmed the presence of near-perfect circular polarization, with DOCP values of 99.2% (left-handed) and 99.5% (right-handed).

These results indicate that the primary cause of the DOCP deviation from 100% is the dichroism/birefringence introduced by optical elements in the detection path, with the dichroic mirror being the main contributor.

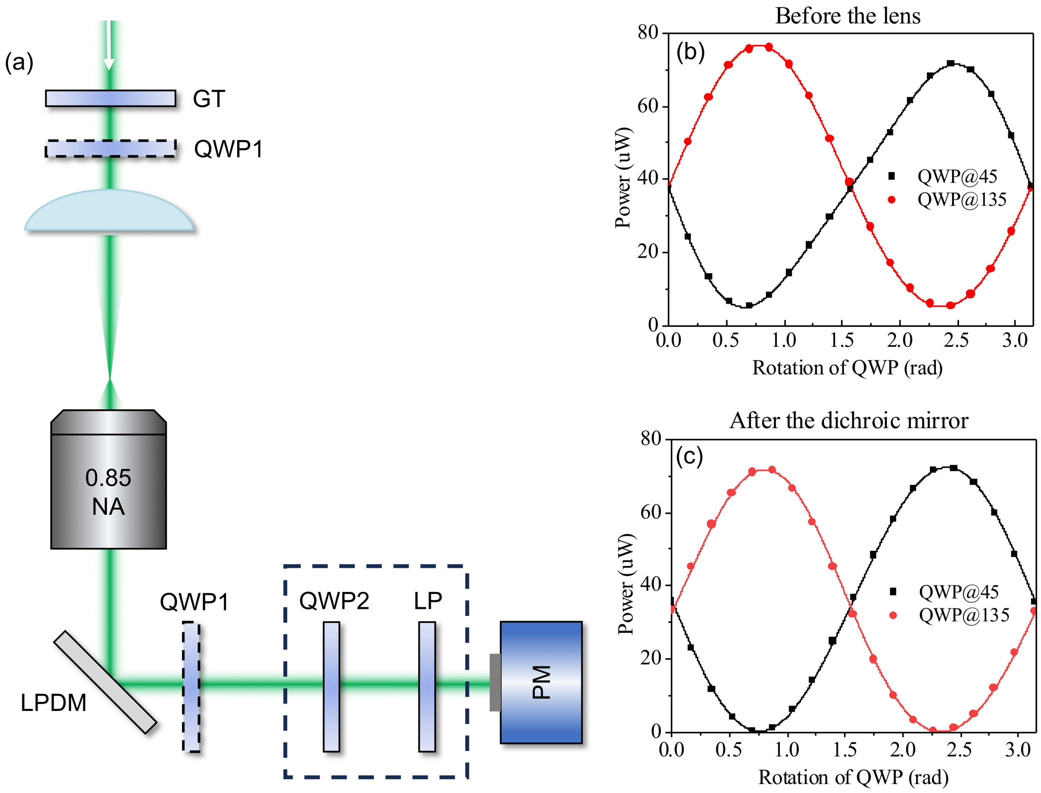

Figure S7: (a) Schematic diagram of the setup used for characterization of polarization dichroism of the collection optics. The green laser centered at 518 nm, corresponding to the wavelength of upconverted light, is sent through the same detection path as in the experimental setup shown in Fig.S3. Power recorded after the polarimetry elements when the quarter-waveplate, creating circular polarization states, (red: right-handed, black: left-handed) is placed (b) before the converging lens and (c) after the dichroic mirror (LPDM). GT: Glan-Taylor polarizer; QWP: Quarter-wave plate; LPDM: long-pass dichroic mirror; LP: linear polarizer; PM: power meter.

S7: Conversion efficiency and coefficient of SFG and THG

The conversion efficiency (η) for SFG and THG is defined as:

$$\eta_{SFG} = \frac{P_{avg}^{SFG}}{\sqrt{P_{avg}^{2\omega} P_{avg}^{\omega}}},$$

$$\eta_{THG} = \frac{P_{avg}^{THG}}{P_{avg}^{\omega}},$$

where P_{avg}^{SFG} and P_{avg}^{THG} represent the time-averaged power of SFG and THG, while P_{avg}^{ω} and $P_{avg}^{2\omega}$ represent the time-averaged power of the fundamental beams at ω and 2ω.

For data analysis, an analytical model of the interference between the two pump pulses[7] is fitted to the interferograms shown in Fig.2. The THG signal is extracted from the baseline (signal far from zero delay) of the fit, while the SFG signal is determined by subtracting the baseline from the peak value of the bell-shape envelope of the fit. In our experiment, the highest conversion efficiencies of the metasurface (*xx* polarization) are $\eta_{SFG} = 1.7 \times 10^{-9}$ and $\eta_{THG} = 7.6 \times 10^{-11}$ using 11 mW and 22 μW average input powers of ω and 2ω beams (peak intensity: $I_{pk}^{\omega} = 0.2$ GW/cm^2, $I_{pk}^{2\omega} = 0.6$ MW/cm^2).

Here we also calculated the conversion coefficient (γ), which is an absolute parameter that solely reflects the nonlinear properties of the sample and does not depend on the input laser intensity, repetition rate, and pulse duration. The conversion coefficient γ for SFG and THG is defined as:

$$\gamma_{SFG} = \frac{P_{pk}^{SFG}}{P_{pk}^{\omega} P_{pk}^{2\omega}},$$

$$\gamma_{THG} = \frac{P_{pk}^{THG}}{(P_{pk}^{\omega})^3},$$

$$P_{pk} = \frac{P_{avg}}{\tau \cdot f_{rep}},$$

where P_{pk} represents the peak power, τ the pulse duration and f_{rep} the repetition

rate.

The conversion coefficients of the metasurface are γ_{SFG} = 7.0 × 10^{-11} W^{-1} for SFG and γ_{THG} = 2.9 × 10^{-16} W^{-2} for THG.

References:

1 Papatryfonos K, Angelova T, Brimont A, Reid B, Guldin S, Smith PR *et al.* Refractive indices of MBE-grown AlxGa(1− x)As ternary alloys in the transparent wavelength region. *AIP Advances* 2021; **11**: 025327.

2 Wang Y, Zilli A, Sztranyovszky Z, Langbein W, Borri P. Quantitative optical microspectroscopy, electron microscopy, and modelling of individual silver nanocubes reveal surface compositional changes at the nanoscale. *Nanoscale Adv* 2020; **2**: 2485–2496.

3 Zilli A, Rocco D, Finazzi M, Di Francescantonio A, Duò L, Gigli C *et al.* Frequency Tripling via Sum-Frequency Generation at the Nanoscale. *ACS Photonics* 2021; **8**: 1175–1182.

4 Yang J, Hugonin J-P, Lalanne P. Near-to-Far Field Transformations for Radiative and Guided Waves. *ACS Photonics* 2016; **3**: 395–402.

5 Collett E. *Polarized Light: Fundamentals and Applications*. Marcel Dekker: New York Basel, 1993.

6 Schaefer B, Collett E, Smyth R, Barrett D, Fraher B. Measuring the Stokes polarization parameters. *American Journal of Physics* 2007; **75**: 163–168.

7 Di Francescantonio A, Locatelli A, Wu X, Zilli A, Feichtner T, Biagioni P *et al.* Coherent Control of the Nonlinear Emission of Single Plasmonic Nanoantennas by Dual-Beam Pumping. *Advanced Optical Materials* 2022; **10**: 2200757.
